# Development and Nationwide Multicentre Evaluation of Guideline-Grounded Large Language Model Chatbots to Support Patient Self-Management and Education in Rheumatology

**DOI:** 10.1007/s10916-026-02470-6

**Published:** 2026-10-05

**Authors:** Tim Wilhelmi, Vanessa Bartsch, Marius Platt, Asarnusch Rashid, Johannes Hornig, Martin Krusche, Axel J Hueber, Daniel Fink, Alexander Pfeil, Gabriel Dischereit, Marie-Therese Holzer, Ulrich Drott, Peer Aries, Max Müller, Peter Böhm, Harriet Morf, Hannah Labinsky, Felix Mühlensiepen, Diego Benavent, Sebastian Kuhn, Philipp Klemm, Johannes Knitza

**Affiliations:** 1 Department of Rheumatology, Clinical Immunology, Osteology and Physical Medicine, Justus Liebig University Giessen, Campus Kerckhoff, Bad Nauheim, Germany; 2 Division of Rheumatology, Paracelsus Medical University, Klinikum Nürnberg, Nürnberg, Germany; 3 School of Medicine, Institute for Digital Medicine, Philipps-Universität Marburg, Marburg, Germany; 4 Zentrum für Telemedizin, Bad Kissingen, Germany; 5 Rheumapraxis an der Hase, Osnabrück, Germany; 6 Division of Rheumatology, III. Department of Medicine, University Medical Center Hamburg-Eppendorf, Hamburg, Germany; 7 Rheumazentrum Mittelhessen, Bad Endbach, Germany; 8 Center of Rheumatology and Section Rheumatology and Osteology, Department of Internal Medicine III, Jena University Hospital – Friedrich Schiller University Jena, Jena, Germany; 9 MVZ Frankenberg, Frankenberg, Germany; 10 Deutsches Endokrinologisches Versorgungszentrum, Frankfurt, Germany; 11 Department of Rheumatology, Immunologikum, Hamburg, Germany; 12 Deutsche Rheuma-Liga e.V, Bonn, Germany; 13 Department of Medicine 3 - Rheumatology and Immunology, Friedrich- Alexander- Universität Erlangen-Nürnberg and Uniklinikum Erlangen, Erlangen, Germany; 14 Department of Internal Medicine 2, Rheumatology/Clinical Immunology, University Hospital Würzburg, Würzburg, Germany; 15 Charité - Universitätsmedizin Berlin, Corporate Member of Freie Universität Berlin, Humboldt Universität zu Berlin, Campus Charité Mitte, Charitéplatz 1, 10117 Berlin, Germany; 16 Rheumatology Department, Hospital Universitari de Bellvitge, Barcelona, Spain

**Keywords:** Rheumatology, Patient education, Large language models, Artificial intelligence, Chatbots, Retrieval-augmented generation

## Abstract

Patients with rheumatic diseases have persistent information needs that are not fully addressed in routine care. We developed and evaluated guideline-grounded, large language model (LLM) chatbots to support patient self-management and education in rheumatology.

Ten disease-specific chatbots based on German guidelines were co-developed and deployed through 13 rheumatology centres and six patient organisations. Chatbot users rated responses and completed a questionnaire. User questions, feedback, and response characteristics were analysed using category-based coding and a six-dimensional LLM-as-a-judge assessment, with LLM-based ratings compared with rheumatologist ratings in random subsets.

Between September 2025 and January 2026, 6291 questions were recorded. Thirteen question categories were identified, most commonly disease-specific questions (50.2%), medication and monitoring (37.3%) and diagnostics (28.2%). The chatbots were unable to answer in 263 interactions (4.2%). Of 2671 responses rated by users, 2481 (92.9%) received a positive rating. Insufficient detail was the most common reason for negative ratings (125/190, 65.8%). Among 602 questionnaire respondents, 84.6% reported that the chatbot was easy to use, 84.1% that answers were easy to understand, and 80.2% that it was a useful addition to patient education. In the LLM-based evaluation, 95.3% of answers were rated as completely safe and 79.1% as completely correct. Guideline adherence was assessed separately, with 45.0% rated as fully adherent; agreement with physician assessment was weak.

Guideline-grounded chatbots received predominantly positive user feedback in real-world use, while LLM-based evaluation suggested that most responses were safe and correct. User questions and feedback may help guide iterative improvements to source content and patient education materials. Further studies are needed to evaluate educational effectiveness and independently validate response quality and clinical safety.

**Supplementary Information:**

The online version contains supplementary material available at https://doi.org/10.1007/s10916-026-02470-6.

## Introduction

Rheumatic diseases are chronic conditions that require lifelong management. Patients must continuously navigate questions related to symptoms, treatment options, side effects, lifestyle adaptations, and disease monitoring. Actively seeking reliable information about one’s own condition is a cornerstone of rheumatology care, supporting shared decision-making, treatment adherence, self-management, and long-term outcomes [1, 2]. Yet many of these questions remain insufficiently addressed in routine care, which is often constrained by limited consultation time [1–3]. Importantly, unanswered questions are not restricted to newly diagnosed patients. Over the course of years and decades, new questions arise as life circumstances change, from pregnancy planning to infection risk. Even patients with longstanding disease may have limited knowledge about disease monitoring and treatment options [4, 5].

Existing patient education resources only partly address this challenge [3]. Leaflets and institutional websites provide structured information but are static and cannot respond to individual, day-to-day questions. Clinical practice guidelines offer the most reliable evidence base, but plain-language patient versions, where available, are often lengthy and difficult to navigate. As a result, many patients seek answers online and increasingly use large language model (LLM)-based chatbots, which provide immediate, conversational access to health information [6–8]. However, while LLMs can generate medically plausible responses that may be perceived as more empathetic and acceptable than physician-derived responses, their accuracy and safety for patient education remain insufficiently validated [9–11]. For use in routine patient self-management and patient education, chatbot responses must be accurate, safe, understandable, and aligned with current clinical guidance.

Retrieval-augmented generation (RAG) can improve reliability by anchoring chatbot responses in predefined source documents rather than relying solely on the model’s internal knowledge [12]. When the retrieval corpus consists of current clinical guidelines, RAG-based systems can combine the accessibility of conversational AI with a controlled evidence base, reducing unsupported and inaccurate statements. However, source anchoring alone is insufficient. Chatbots must also provide understandable and relevant answers. The recently reported LupusGPT initiative provides an important patient-led example of a retrieval-augmented, multilingual chatbot for systemic lupus erythematosus [8]. However, evidence in this field remains limited, particularly regarding other diseases and structured evaluations that combine real-world use, patient acceptance, and systematic assessment of response quality and safety.

In this prospective, multicentre, mixed-methods study, we developed and evaluated ten disease-specific, guideline-grounded LLM chatbots for real-world use in rheumatology. We aimed to characterise chatbot usage patterns and user perceptions, analyse the content of user questions and feedback, and assess response quality, safety, and guideline adherence.

## Methods

This nationwide multicentre study was conducted in Germany and is reported in accordance with the CHART guideline (Supplementary Checklist S1). The project involved patient research partners, patient organisations, rheumatologists, health services researchers and technical experts in a collaborative approach. Two official patient research partners from Deutsche Rheuma-Liga e.V., the largest German patient organisation, were nominated by the organisation following our request and involved throughout the project. They contributed to chatbot development and refinement, including source document selection and optimisation of system prompts, as well as to study design, questionnaire development, dissemination, analysis and reporting. Their input led to more empathetic language in the system prompts, clearer questionnaire wording, a simplified disclaimer and refinements to the category labels and definitions used in the qualitative analysis. They also advised on dissemination. Both partners received training through Deutsche Rheuma-Liga e.V. and were not financially compensated. Collaboration took place entirely online, and selection did not follow a documented criteria-based process. Patient involvement is further documented in the GRIPP2 short-form checklist (Supplementary Checklist S2). The Ethics Committee of Philipps University Marburg classified the study as exempt from ethical approval (reference: 25-237-Anz). Participation was voluntary, and participants were informed that study data would be used for research purposes. Patients were informed that the chatbot could produce erroneous responses, should not be used as a substitute for professional medical advice and that no personal data should be entered. Chatbot access was intended for adult rheumatic patients, including those presenting for initial diagnostic work-up.

### Chatbot Architecture

The chatbots were developed using a commercial platform (Buddy; Zentrum für Telemedizin (ZTM) Bad Kissingen GmbH, Germany; https://www.ztm.de/buddy/) and accessed OpenAI’s GPT-4o model (version 2024-11-20) via an Azure OpenAI endpoint deployed in Germany. The model was not fine-tuned, with behaviour controlled entirely by retrieval-augmented generation and the system prompt. User inputs and AI-generated responses were not used for foundation-model training. All study data were processed and stored on dedicated ZTM infrastructure in Karlsruhe, Germany. No user accounts or direct identifiers were collected. Conversations were linked using randomly generated identifiers. Free-text entries were not automatically de-identified and could therefore contain identifying information despite instructions not to enter personal data. Conversations were stored in the application database and LLM gateway without an automated deletion routine during the study. For each chatbot, the knowledge base was limited to the latest available valid German clinical guideline for the respective rheumatic disease (Table S6). Guideline content was hierarchically segmented and embedded in a vector database using a fine-tuned, domain-specific, large-context embedding model. Candidate passages were retrieved from hierarchically chunked guideline sections using hybrid lexical and dense-vector retrieval and ranked using a statistical reranker weighting vector and token similarity 70%/30% (a semantic reranker was tested but not adopted); passages below a 20% similarity threshold were excluded, and up to eight were inserted into the prompt per turn together with source identifiers, which were displayed to users alongside chatbot responses. In multi-turn conversations, queries were resolved against the preceding dialogue and rewritten as stand-alone queries before retrieval, and source citations were assigned in a separate post-generation step matching answer sentences to the retrieved supporting passages. To favour controlled and reproducible outputs, the response style was set to ‘precision’: a preset bundling temperature and top-p, superseded once these were set manually to 0.10 and 0.30 respectively. The system prompt instructed the model to answer patient questions about rheumatic diseases in plain language, avoid complex medical terminology and unexplained abbreviations, and adhere strictly to the guideline-based knowledge base. Guidance to seek professional advice for medication changes or urgent medical decisions was included in the system prompt but was not enforced by a deterministic clinical safety mechanism. Azure’s default harmful-content filters were general content filters, not clinical safeguards for medication- or urgency-related queries. No real-time clinical safety monitoring or escalation pathway was implemented.

### Dissemination

The chatbots were available continuously throughout the study period, from 2 September 2025 to 31 January 2026. Access links were disseminated by participating rheumatologists at 13 centres in Germany using disease-specific leaflets and posters containing QR codes and brief instructions for use. Access was also promoted through rheumatology patient organisations, patient research partners and advocacy groups, including newsletters and patient-oriented social media channels.

### Evaluation

Chatbot usage patterns were characterised using the number of interactions and sessions, conversation depth, timing of use and feedback rates. User perceptions were assessed through structured response ratings, free-text feedback and an optional evaluation questionnaire. A session was defined as a continuous chatbot interaction, and a turn as one question–answer exchange within a session. Conversation depth was defined as the number of turns per session. After each chatbot response, users could provide an immediate binary rating (“like” or “dislike”) and, optionally, free-text feedback. For negatively rated responses, users were asked to select the main reason from predefined categories: factual inaccuracy, unsafe, harmful or illegal content, offensive, discriminatory or unfair language, repetitive output, insufficient clarity, lack of detail, or other. Each chatbot response also included a link to an optional evaluation survey (see Supplementary Survey S1). The survey collected information on diagnosis, age, previous use of AI-based tools for health-related questions and perceived role of AI in healthcare. Additional items, assessed on 5-point Likert scales, covered ease of use, comprehensibility of chatbot responses, perceived usefulness as a supplement to existing patient education materials, expected time savings when searching for information, answer quality, trustworthiness and preference for the chatbot over general internet searches, such as Google. The survey also included a free-text field for suggestions to improve the chatbot. The content of user questions and feedback was analysed separately using qualitative content analysis, while response quality, safety and guideline adherence were assessed using the LLM-based evaluation approach described below.

### Qualitative Coding of Questions and Feedback

For expert-guided analysis at scale, we tested a rheumatologist-guided, human-in-the-loop local LLM-as-a-judge approach for qualitative content analysis with inductive category development [13]. LLM analysis was performed using a locally hosted, data secure gpt-oss:120b model deployed via Ollama with a temperature setting of 0. For initial category development, random samples of 100 user questions, 100 free-text comments on chatbot responses and 100 free-text comments from evaluation questionnaires were selected. A multistage human-in-the-loop verification process was then used to review and refine the LLM-generated categories before applying the final coding framework to the full dataset [14]. The predefined prompt used for category development is provided in Supplementary Method S1. To support stable processing, each set of 100 items was divided into five batches of 20 items. No additional categories emerged in the final batches, indicating that the sample size was sufficient. A rheumatologist then reviewed the LLM-generated category suggestions and used them to categorise the respective examples. During this review, overlapping categories were merged and category labels and definitions were refined. Coding was multi-label: a question or comment could be assigned to more than one category if it addressed several topics. These physician-coded examples were subsequently used to refine few-shot prompts for automated coding. The final prompt used for automated coding and quality evaluation is provided in Supplementary Method S2. After prompt refinement, the LLM was used to classify all patient questions and feedback comments using a few-shot system prompt. To mitigate potential bias related to category order, three independent coding runs were performed. Examples presented in the manuscript are derived from actual user questions and comments and were translated from German into English.

### Question Difficulty and Chatbot Answer Quality

The rheumatologist-guided LLM-as-a-judge approach was also investigated to assess question difficulty and chatbot answer quality. Ratings were based on six dimensions adapted from the framework by Hao et al.: answer correctness, patient question difficulty, answer completeness, patient-readiness, safety and patient-centredness [15], all assessed as evaluator ratings rather than patients’ own reports. Each dimension was rated on a 3-point ordinal scale, with 1 representing the lowest and 3 the highest rating. Exact rating definitions for each dimension are provided in Supplementary Method S2. Two board-certified rheumatologists, each with more than 10 years of clinical experience, independently evaluated 100 randomly selected chatbot question–answer pairs for question difficulty and answer quality dimensions, blinded to each other’s ratings. The automated LLM-based ratings were generated only after this physician evaluation was completed. These physician ratings were used to refine few-shot system prompts for automated LLM-based assessment and, in addition, to assess inter-rater agreement between the two physicians as a benchmark for LLM-physician agreement (Table S3). For the full dataset, each question–answer pair was evaluated separately for each quality dimension and for guideline adherence using independent LLM invocations. Clinical correctness was assessed independently of source provenance, whereas guideline adherence assessed whether the response was supported by the guideline passages retrieved for that interaction.

### Statistical Analysis

Descriptive statistics were used to summarise chatbot usage, user ratings, survey responses, physician and LLM-based evaluation results. Continuous variables were summarised using means and standard deviations or medians and interquartile ranges, as appropriate; categorical variables were summarised using frequencies and percentages.

For qualitative coding, agreement between physician-coded and LLM-coded classifications was assessed using percentage agreement and Gwet’s AC1 [16]. Agreement was evaluated in random samples of 50 user questions, 50 free-text comments on chatbot responses and 100 free-text comments from evaluation questionnaires. Gwet’s AC1 was selected as the primary chance-corrected agreement coefficient because Cohen’s κ is susceptible to the so-called kappa paradox, in which low κ values may occur despite high observed agreement when category prevalence is very low or very high [17]. This was relevant because several coding categories showed markedly imbalanced distributions.

For question difficulty and the five chatbot-quality dimensions, ordinal agreement was assessed in the 100 physician-rated question–answer pairs using weighted Gwet’s AC2, separately for the LLM versus each rheumatologist and between the two rheumatologists. Agreement for guideline adherence was assessed separately against one rheumatologist [18]. The two physician ratings were retained independently. No composite or adjudicated physician rating was constructed. Separately from AC2, percentage agreement with either physician was calculated as the proportion of LLM ratings matching at least one physician’s rating. Gwet’s AC2 was selected because Cohen’s κ can systematically underestimate agreement in the presence of skewed marginal distributions [17]. Agreement was interpreted according to Landis and Koch, with values ≥ 0.60 considered substantial, 0.40–0.59 moderate, and < 0.40 weak [19]. Systematic differences were assessed using the Wilcoxon signed-rank test for each LLM–rheumatologist comparison across question difficulty and the five chatbot-quality dimensions (safety, patient-centredness, correctness, completeness, patient-readiness). Guideline adherence was not included in this test family due to its low baseline agreement. To account for the resulting twelve comparisons (six dimensions × two physicians), Bonferroni correction was applied, and the adjusted p-value for each comparison is reported separately. All statistical and descriptive analyses were conducted using R version 4.3.

## Results

### Chatbot Usage Patterns

**Fig. 1:**
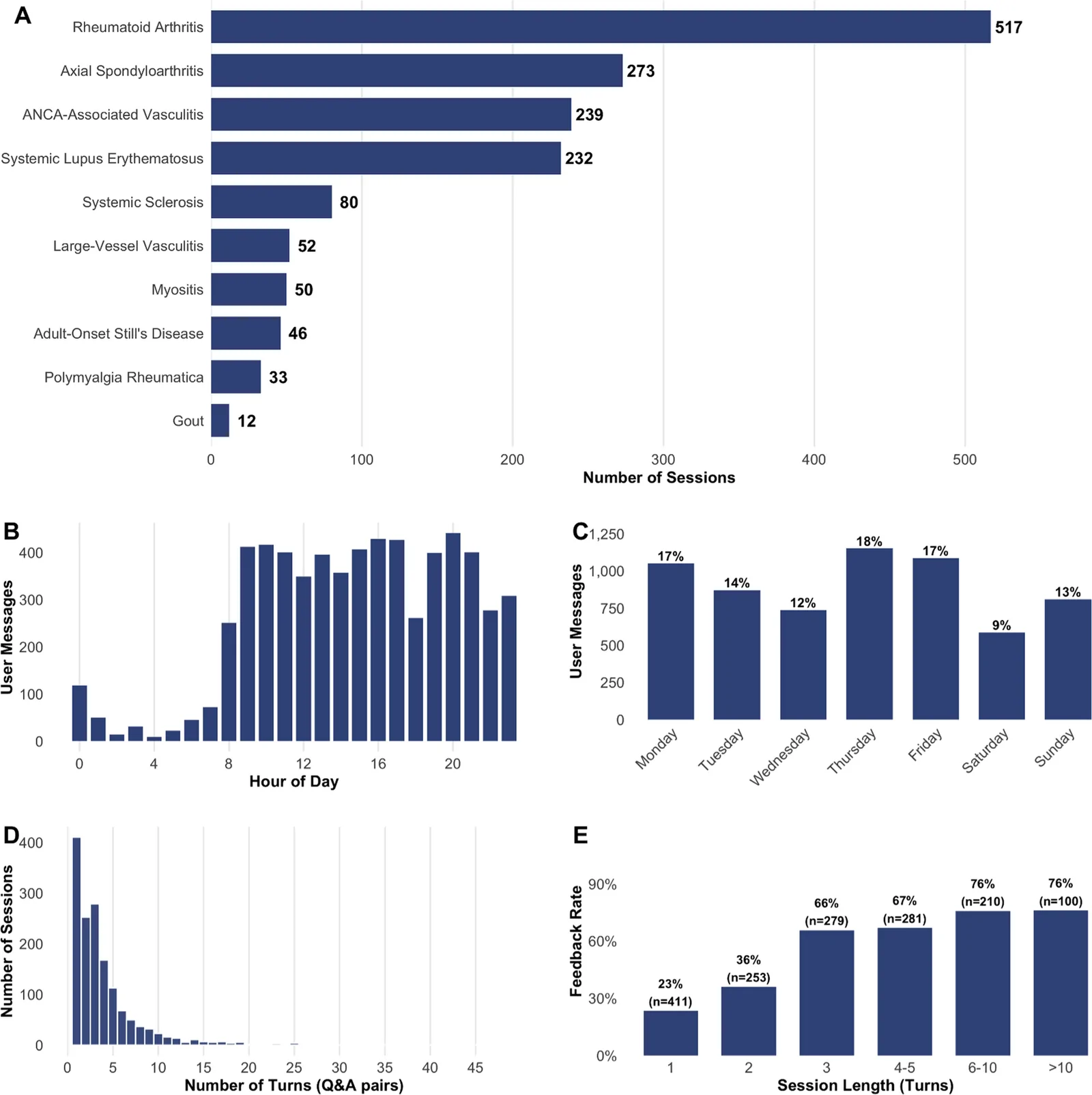
Chatbot usage patterns. (**A**) Distribution of chatbot sessions across ten disease-specific chatbots. (**B**) Hourly distribution of user messages showing peak usage during daytime and evening hours. (**C**) Weekly distribution showing higher engagement on weekdays compared to weekends. (**D**) Distribution of conversation depth measured as number of question-answer pairs (turns) per session. The red dashed line indicates the median. (**E**) Feedback rate by session length, showing that longer sessions had higher proportions of users providing ratings

Between 2 September 2025 and 31 January 2026, a total of 6291 chatbot interactions (question–answer pairs) were recorded across 1534 individual chatbot sessions. In 4.2% (263/6291) of cases, the chatbot indicated that it could not answer the question. Rheumatologist review of these cases found that 17.1% (45/263) would have been answerable using the respective guideline. Chatbots for rheumatoid arthritis (RA; *n* = 517 sessions), followed by axial spondyloarthritis (axSpA; *n* = 273) and ANCA-associated vasculitis (AAV; *n* = 239), were most often used, see Fig. 1. Analysis of hourly distribution showed highest engagement during daytime hours, with substantial usage extending into evening hours. Weekly patterns indicated peak usage mid-week, with reduced engagement on weekends. The average conversation depth was 4.1 turns per session (median: 3). Longer sessions showed higher feedback rates.

**Fig. 2:**
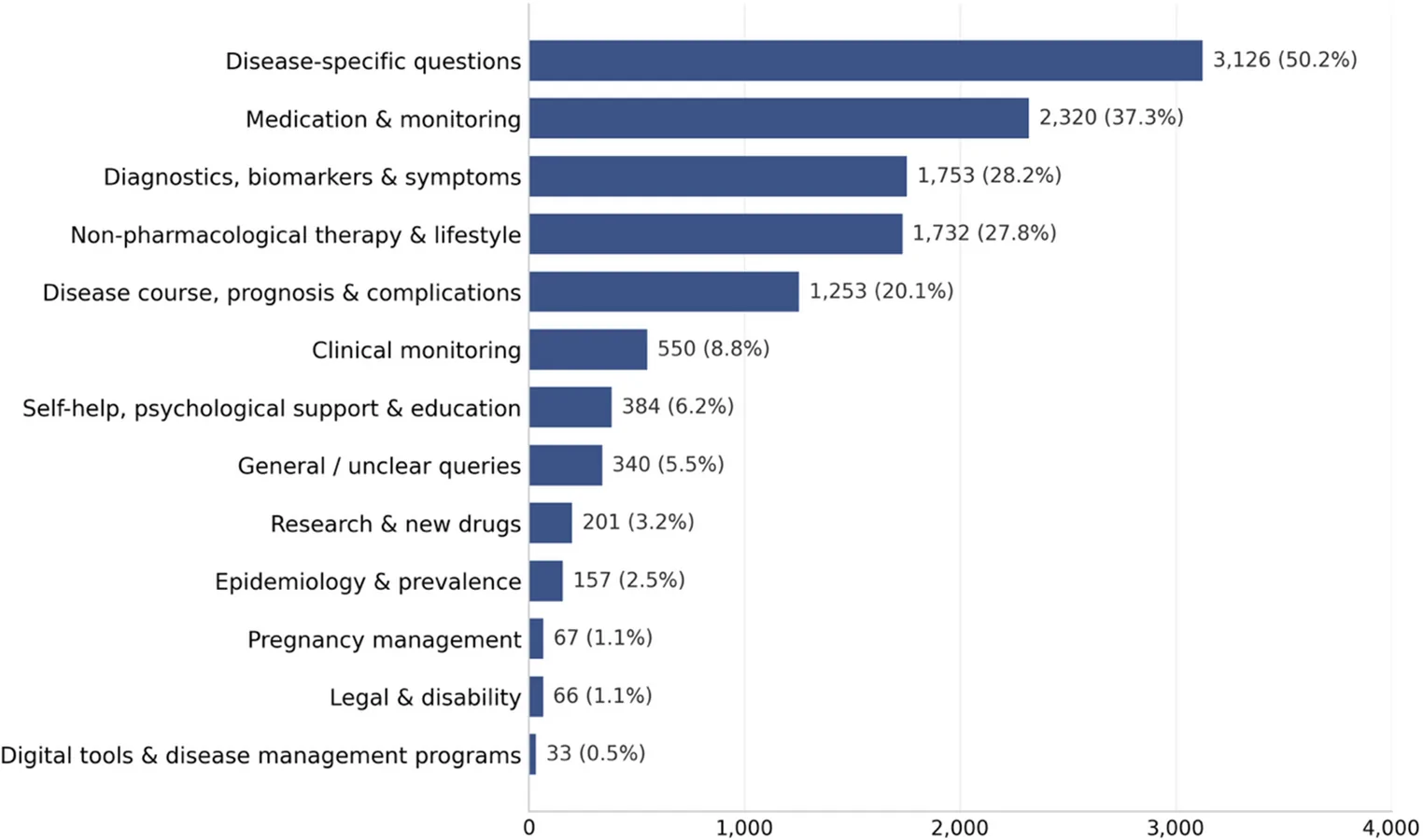
Prevalence of patient question categories. Horizontal bar chart showing the prevalence of categories identified among patient questions. Bars indicate the absolute number of questions assigned to each category, with percentages representing the proportion of all questions. As multi-label coding was permitted, percentages sum to more than 100%

Most common topics were disease-specific questions (50.2%, example: “How can I prepare for a doctor’s appointment to better discuss my vasculitis?“), followed by medication and monitoring (37.3%, example: “When do the medications start working? I have been taking them for 1.5 weeks. The symptoms are already better, but not completely gone yet.”), diagnostics (28.2%, example: “Which clinical symptoms indicate a GPA flare when no serological markers are present?“), non-pharmacological interventions and lifestyle (27.8%, example: “Is strength training or endurance exercise better for my back?“), and prognosis and complications (20.1%, example: “How long does it usually take until the disease is under control for the first time?“), see Fig. 2. LLM-based coding showed strong agreement with human coding, with a mean concordance of 93.6%. Gwet’s prevalence-robust AC1 coefficient averaged 0.91.

### User Perceptions of Chatbot Responses

Of all chatbot responses, 42.5% (2671/6291) were rated by users, of which 92.9% (2,481/2671) received a “like” and 7.1% (190/2671) a “dislike”. Positive ratings substantially outnumbered negative ratings across all chatbots. The most common reason for a dislike was “insufficient” 65.8% (125/190), as shown in Fig. S1. Most chatbot-response free text comments concerned response quality and clinical accuracy (42.7%, example: “Professionally accurate answer; a paragraph on MRI vs. CT would have been good.“), followed by treatment options and pharmacology (18.8%), evidence, sources and data quality (10.0%, example: “Rinvoq and Xeljanz are approved for ankylosing spondylitis”), diagnostic and symptom context (10.0%, example: “I would also have expected the paranasal sinuses”), and resources and interaction wishes (9.2%, example: “Sufficient, but further links would be helpful”), see Fig. S2. Automated LLM coding demonstrated substantial agreement with human coding, achieving an average concordance of 88.8%. Mean Gwet’s prevalence-robust AC1 coefficient was 0.85.

### Questionnaire-Based User Perceptions

**Fig. 3:**
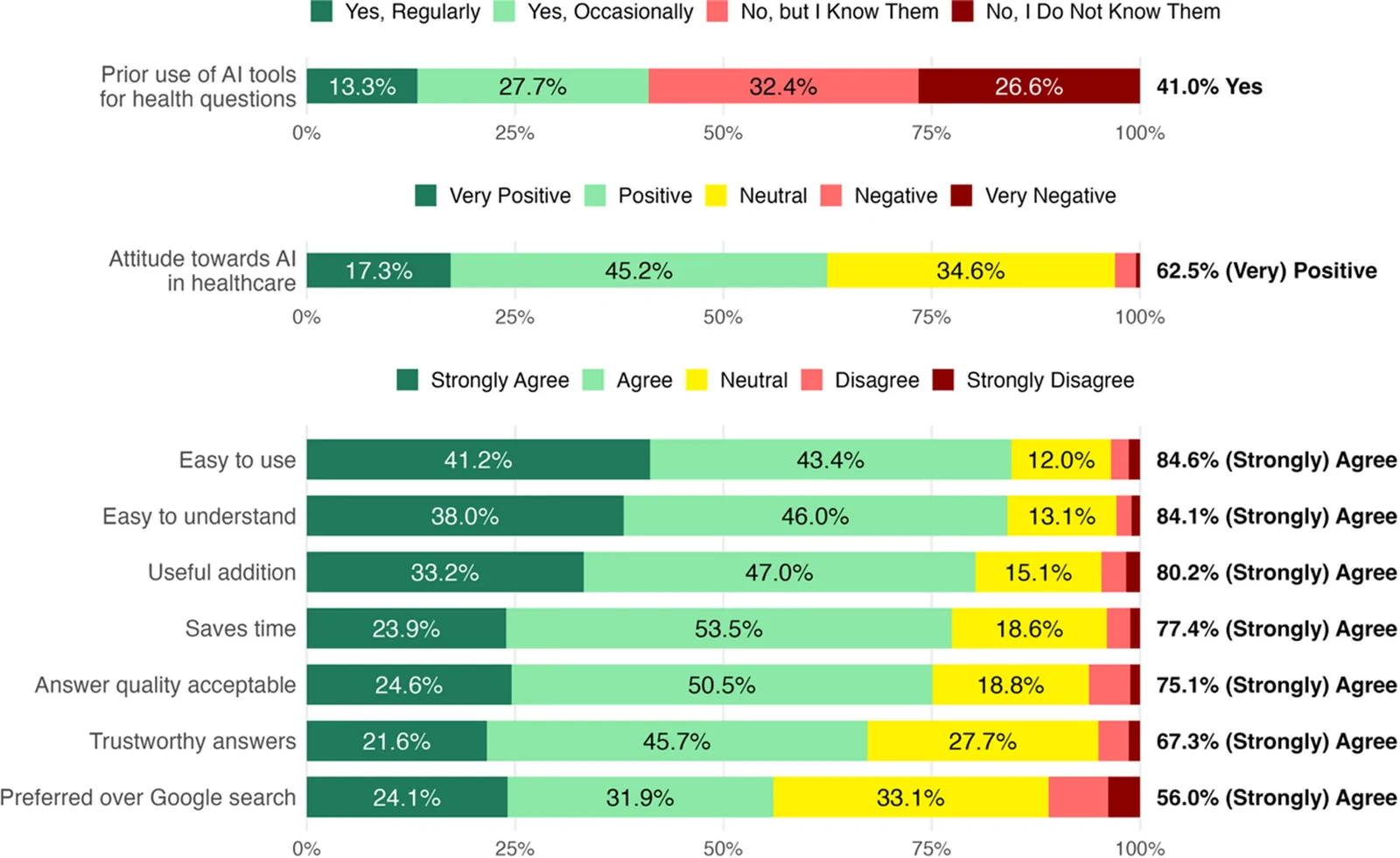
Results of chatbot evaluation questionnaire. Responses are shown as proportional stacked bar charts and presented as proportions across four Likert-scale categories (Yes, regularly to no I don’t know them) or five Likert-scale categories (strongly agree to strongly disagree and very positive to very negative)

A total of 602 users (Table 1) completed the optional study-specific evaluation questionnaire. Among questionnaire respondents, 93.0% (560/602) reported a diagnosed IRD, most commonly RA (*n* = 234), axSpA (*n* = 111), and AAV (*n* = 75). The mean (SD) age was 49.8 (13.7) years. Previous use of AI-based tools for health-related questions was reported by 41.0% (247/602) of participants. 62.5% (376/602) perceived AI in healthcare as positive or very positive, 34.6% (208/602) as neutral and 2.9% (18/602) as negative or very negative, see Fig. 3. Overall, 84.6% (strongly) agreed that the chatbot was easy to use and 84.1% that its answers were easy to understand, while 80.2% considered it a useful addition to existing patient education materials. Furthermore, 77.4% indicated that the chatbot would save time when searching for answers, 75.1% rated the answer quality as at least acceptable, 67.3% found the answers trustworthy, and 56.0% preferred using the chatbot over general internet searches (e.g. Google).\

**Table 1:** Characteristics of users who completed the evaluation questionnaire

Characteristic		Overall (*N* = 602)
Age, mean (SD)		49.8 ± 13.7
Sex, n (%)		
	Female	461 (76.6%)
	Male	141 (23.4%)
Diagnosis, n (%)		
	Rheumatoid Arthritis	234 (38.9%)
	Axial Spondyloarthritis	111 (18.4%)
	ANCA-Associated Vasculitis	75 (12.5%)
	Systemic Lupus Erythematosus	51 (8.5%)
	No confirmed rheumatological diagnosis	42 (7.0%)
	Adult-Onset Still’s Disease	21 (3.5%)
	Systemic Sclerosis	21 (3.5%)
	Myositis	18 (3.0%)
	Large-Vessel Vasculitis (including Giant Cell Arteritis)	16 (2.7%)
	Polymyalgia Rheumatica	9 (1.5%)
	Gout	4 (0.7%)

*ANCA* anti-neutrophil cytoplasmic antibodies, *SD* standard deviation

### Free-text Feedback

Most free-text survey comments concerned response quality and depth (28.6%, example: “I would have appreciated a more detailed answer to my nutrition question regarding omega-3 fatty acids: which ones are important, what dose, and which options besides fish are there?“), followed by language and clarity (19.3%, example: “The answers are very dry; ChatGPT’s voice mode feels less professional but more empathetic, this could be adjusted”), sources and transparency (17.9%, example: “I would appreciate tips on where to find further reputable information on the respective topic”), content coverage and expansion (14.3%, example: “For such a multifaceted disease there should be more cross-links between topics”), and trust and reliability (10.7%, example: “Build more trust by providing fuller source details and longer answers.“), see Fig. S3. LLM coding achieved a high degree of agreement (mean 97.3%) with human coding. Gwet’s prevalence-robust AC1 coefficient (mean 0.96) indicated high chance-corrected agreement with human coding.

### Evaluation of Question Difficulty and Chatbot Response Quality

**Fig. 4:**
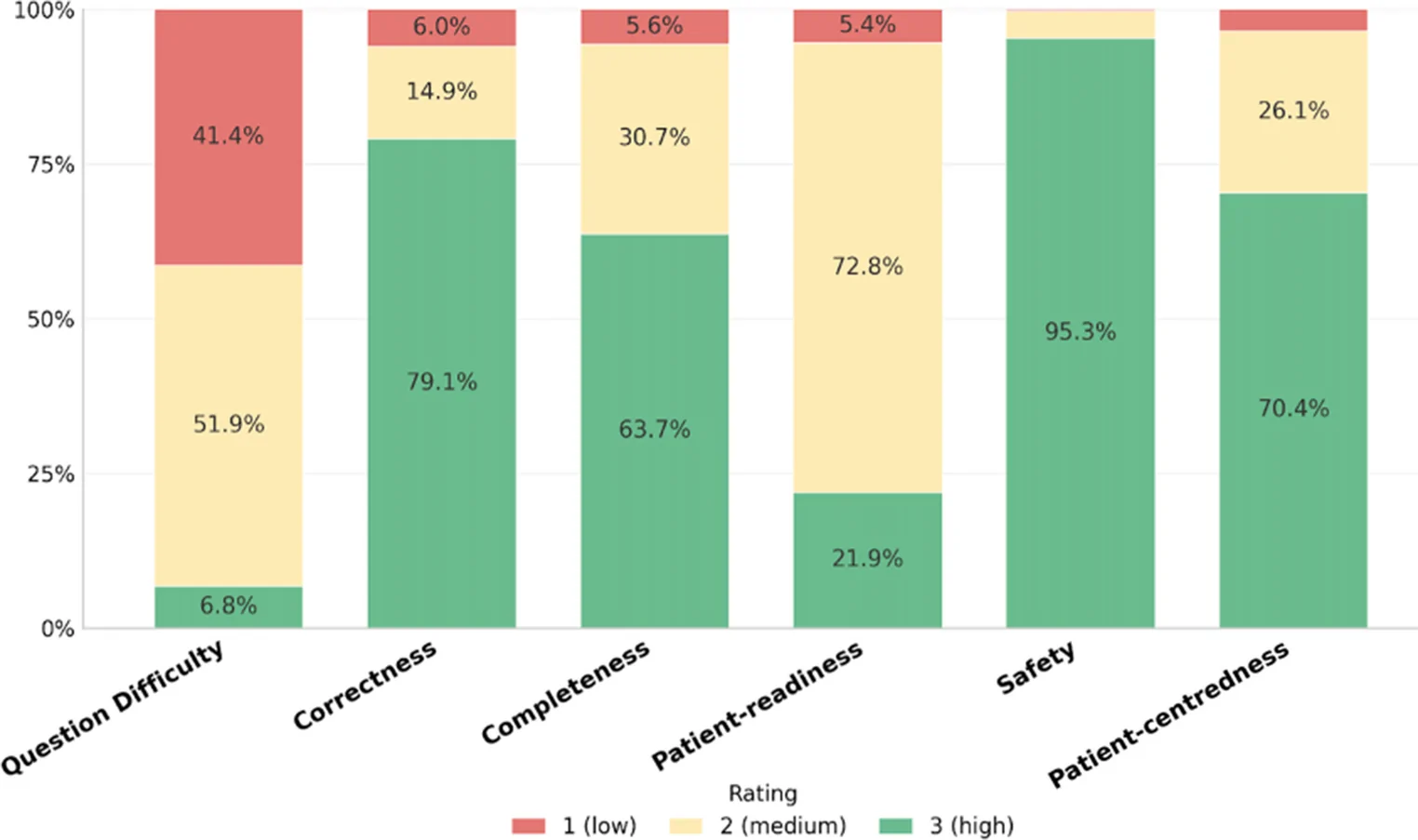
Large language model-based evaluation of chatbot question–answer pairs**.** Distribution of ratings across six evaluation dimensions for 6291 chatbot question–answer pairs. Each dimension was rated on a 3-point ordinal scale, with 1 indicating the lowest and 3 the highest rating. Safety, correctness, patient-centredness, and completeness were the dimensions for which most responses received high ratings

LLM-based ratings for all 6291 question–response pairs across the six evaluation dimensions are shown in Fig. 4. Detailed results are shown in Table S1. The proportion of responses receiving the highest rating varied considerably across dimensions: safety (95.3%), overall correctness (79.1%; 14.9% partially correct, reflecting minor or outdated inaccuracies, and 6.0% incorrect, reflecting major errors with potential for harm), patient-centredness (70.4%), completeness (63.7%), patient-readiness (21.9%) and question difficulty (6.8%). Chatbot answer correctness was sometimes affected by strict adherence to the uploaded guideline documents, as these did not always reflect the most recent drug approvals. For example, one response stated: “At present, no Janus kinase inhibitors (JAK inhibitors) are specifically approved for the treatment of Bechterew’s disease (ankylosing spondylitis),” reflecting the guideline content available to the chatbot rather than the current regulatory status. Guideline adherence was rated separately. 45.0% (2,828/6,291) of responses were rated as fully guideline adherent, 44.2% (2,779/6,291) as partially adherent, and 10.9% (684/6,291) as not adherent. Given the weak agreement with physician assessment (AC2 = 0.38 [95% CI 0.21, 0.53]), these LLM-based estimates should be interpreted cautiously.

The LLM classified 0.2% of chatbot answers (11/6,291) as potentially unsafe. On subsequent physician review, both rheumatologists rated one of these 11 answers as potentially unsafe. The individual safety ratings are provided in Table S5. In this case, the chatbot stated that cortisone could be administered subcutaneously during disease flares. The distribution of human and LLM ratings for the validation subsets is shown in Table S2. Agreement was highest for safety (AC2: 0.91 and 0.96 for rheumatologists 1 and 2, respectively), followed by patient-centredness (0.87 and 0.82), overall correctness (0.82 and 0.74) and completeness (0.81 and 0.68; Table S4; Fig. S4). For patient-readiness, agreement was moderate (AC2: 0.48 and 0.50) with significant systematic differences in both comparisons (adjusted *p*<.001 for each). The LLM rated substantially fewer responses as fully patient-ready (21.9%) than rheumatologists 1 and 2 (93% and 86%, respectively). Agreement for question difficulty was weak (AC2: 0.15 and 0.19), with significant systematic differences in both comparisons (adjusted *p*<.001 for each). For patient-centredness, a significant systematic difference was observed with rheumatologist 1 (adjusted *p*<.001), but not rheumatologist 2 (adjusted *p* = .150). No significant systematic differences were detected for safety, overall correctness or completeness. As a benchmark, agreement between the two physicians themselves was substantial to almost perfect across all six dimensions (AC2 0.60–0.97; Table S3), indicating that LLM-physician agreement for patient-readiness and question difficulty remained below the level of agreement observed between physicians.

## Discussion

To our knowledge, this is the first study to evaluate user perceptions, response quality, and safety of disease-specific, guideline-grounded chatbots for rheumatic diseases in real-world use. The large proportion of positive response ratings and favourable questionnaire results indicate generally positive feedback, while rheumatologist-guided automated evaluation suggested that chatbot answers were predominantly safe and correct. These findings support guideline-based chatbots as a useful complement to support patient self-management and education in rheumatology.

Our findings extend prior work such as LupusGPT [8], which demonstrated broad international use of a retrieval-augmented chatbot for systemic lupus erythematosus. However, LupusGPT was limited to a single rheumatic disease and did not report systematic patient acceptance data or structured response quality and safety assessment.

Chatbot use extended into evening hours and weekends, consistent with evidence that patients predominantly use digital health tools outside of business hours, underscoring the value of immediate, low-barrier access to reliable information independent of consultation schedules [20, 21]. The questions submitted by users were not limited to generic disease definitions but often concerned practical issues that arise between consultations, such as how treatments are monitored and what to expect from medication. These topics reflect previously documented unmet information needs in rheumatology [1, 3]. The median conversation length of three turns suggests that most interactions were brief and focused, with users seeking targeted answers rather than extended consultations. Unlike leaflets or static websites, a conversational system enables these highly personalised answers. This positions guideline-grounded chatbots as accessible complementary tools, rather than a replacement for structured education programmes or clinician-led shared decision-making [22, 23].

Of rated responses, 92.9% received positive feedback, and most questionnaire respondents considered the chatbot easy to use, easy to understand and a useful addition to existing patient education. Notably, 56% of users preferred the chatbot over general internet searches, consistent with evidence that patients value synthesised, direct answers that reduce information overload and search fatigue [24]. However, trustworthiness was endorsed by only 67.3% of respondents. This aligns with broader findings that trust in AI-generated health information depends not only on accuracy but also on perceived utility, social endorsement and institutional credibility [25, 26]. Insufficient detail was the most common reason for negative ratings, and free-text feedback frequently called for greater depth and broader content coverage, suggesting that guideline-only knowledge bases may need to be supplemented with additional verified sources. Relatedly, the chatbot indicated that it was unable to answer in 263 interactions (4.2%), of which rheumatologist review found that most refusals reflected true knowledge-base limitations rather than retrieval failures. In some cases, responses reflected uploaded guideline content that did not yet incorporate recent drug approvals. In other cases, the chatbot provided clinically appropriate answers that drew on its general training knowledge rather than the curated guideline corpus, despite system prompt instructions and conservative generation settings (temperature 0.1, top-p 0.3). Similar limitations have been reported for other RAG-based medical chatbots with restricted document corpora [27]. This illustrates a fundamental tension in guideline-grounded chatbot design: responses beyond the curated knowledge base undermine source transparency, while strictly limiting output to a single guideline may leave relevant patient questions unanswered, reducing user trust [28]. Addressing this trade-off requires refined retrieval configuration and, where appropriate, controlled integration of additional verified content. Only 45.0% of responses were fully guideline-adherent.

The safety and correctness findings are consistent with emerging evidence that RAG-based approaches improve clinical accuracy and reduce hallucinations compared with unconstrained models [29–32]. Rheumatologist-guided LLM ratings indicated that 95.3% of chatbot answers were rated as completely safe, 79.1% as completely correct and 70.4% as highly patient-centred. For comparison, a recent red-teaming study of publicly available, non-RAG chatbots reported unsafe response rates of 5–13% depending on the model, substantially higher than the rate observed in the present study [33]. However, naturalistic and adversarial evaluation conditions differ substantially, and the lower unsafe-response rate observed here should not be interpreted as evidence of superior safety. Among answers flagged by the automated evaluation as potentially unsafe, subsequent physician review confirmed one clearly potentially unsafe response. Given the rarity of such events, these findings should be interpreted cautiously and do not establish reliable detection of rare safety failures, underscoring the need for dedicated safety evaluation and human oversight.

A further contribution of this study is methodological, building on previous LLM-as-a-judge approaches in rheumatology [31]. The rheumatologist-guided automated LLM approach enabled category-based coding and structured quality evaluation across thousands of interactions, consistent with recent evidence that LLM-based evaluation can approximate expert assessment at a fraction of the time [34, 35]. Agreement with physician-coded validation samples was substantial for safety, patient-centredness, correctness and completeness, moderate for patient-readiness, and weak for guideline adherence and question difficulty, indicating that not all evaluative tasks are equally suitable for automated assessment. For patient-readiness and question difficulty specifically, the LLM rated substantially fewer responses at the highest level than either rheumatologist, who agreed closely with each other on both dimensions (Table S3). This suggests that the LLM-based patient-readiness estimate of 21.9% was substantially more conservative than the physician ratings and may contribute to the contrast with the higher user-reported comprehensibility (84.1%). This pattern is consistent with reported central tendency bias in LLM-based ordinal scoring, where models disproportionately avoid extreme ratings compared to human raters [36, 37]. LLM-assisted evaluation should therefore be viewed as a scalable screening and analytic tool, not as a substitute for expert validation.

Several limitations should be considered. Chatbot use was voluntary and may therefore have preferentially attracted individuals with greater interest in health information or digital tools. In addition, only 42.5% of chatbot responses received a rating, and completion of the evaluation questionnaire was optional, introducing further responder bias. Because no user accounts or direct identifiers were collected, repeated sessions by the same individual cannot be excluded, and the clinical status of individual users could not be verified. Diagnoses reported by questionnaire respondents were self-reported and were not independently confirmed. Ultimately, patient outcomes were not analysed and future studies should assess whether chatbot use improves knowledge, self-efficacy, treatment adherence or health service use. Importantly, patients, like physicians, should also rate the responses to evaluate the LLM-as-a-judge approach, particularly regarding patient-centredness. Not all responses were manually reviewed by rheumatologists, and automated evaluation depends on model choice, prompt design and calibration against limited validation samples. Because the validation sample was randomly selected rather than enriched for unsafe, incorrect, or medication-related responses, the study provides limited evidence regarding the ability of the LLM-as-a-judge approach to detect rare safety-critical failures. Dedicated evaluation in an enriched safety-focused sample is therefore needed. Guideline-adherence ratings were validated against a single rheumatologist rater only. Beyond Azure’s default content filters (not specific to medication or urgency) and the system-prompt instruction to seek professional advice, no real-time escalation or safety-monitoring mechanism was implemented. The lack of standardised reporting frameworks for chatbot evaluation studies, as recently highlighted, further limits comparability across studies [38]. Finally, the exclusive grounding in German clinical guidelines supported transparency but limited answer coverage for topics not addressed or not fully up to date in the guidelines.

## Conclusion

Disease-specific, guideline-grounded chatbots were successfully deployed across a nationwide rheumatology network, and received predominantly positive feedback from users who rated responses or completed the questionnaire. LLM-based evaluation suggested that most evaluated responses were safe and correct. User questions enabled insights to improve patient education material. By offering immediate, low-barrier access to reliable information at any time, such systems may accompany patients through evolving disease phases, addressing information needs that routine care alone cannot fully meet. Realising this potential at scale requires robust source governance, more timely guideline updates, integration of additional verified patient information materials, transparent answer boundaries, continuous safety monitoring and sustained human oversight.

## Supplementary Information

Below is the link to the electronic supplementary material.

Supplementary Material 1

## Data Availability

De-identified aggregated study data, the data dictionary, study protocol, and statistical analysis plan will be made available by the corresponding author upon reasonable request from the date of publication. Requests will be considered for scientifically sound research proposals and are subject to approval by the study investigators.

## Code Availability

Custom analysis scripts are available from the corresponding author upon reasonable request.

## Declarations

### AI Declaration

During the preparation of this manuscript, the authors used ChatGPT (OpenAI) and Claude (Anthropic) to assist with language editing and text refinement. All AI-generated output was critically reviewed, verified, and revised by the authors, who take full responsibility for the final content of the manuscript. Large language models were also used as part of the study methodology, as described in the Methods section.
